# Study on Graphene-Reinforced Epoxy Solvent-Borne High-Temperature-Resistant Adhesives for Bonding C/C Composites Under Extreme Temperatures

**DOI:** 10.3390/ma18174213

**Published:** 2025-09-08

**Authors:** Yue Wang, Yuqing Zhang, Zhanming Hu, Jingjing Li, Zhuo Gao, Mingchao Wang, Haijun Zhang

**Affiliations:** 1 College of Science, Civil Aviation University of China, Tianjin 300300, China; yuewang5026@163.com (Y.W.); zhanminghu28@163.com (Z.H.); ljjzj2018@163.com (J.L.); zhuogaocauc23@163.com (Z.G.); 2 School of Electronic Science and Engineering, Southeast University, Nanjing 211189, China; yuqingz0612@foxmail.com; 3 School of Safety Science and Engineering, Civil Aviation University of China, Tianjin 300300, China

**Keywords:** bionic nacre structure, epoxy solvent-borne adhesive, graphene, carbon nanotubes, brick-and-mortar, bonding strength

## Abstract

Drawing inspiration from the bionic nacre structure, graphene was incorporated into the epoxy solvent-borne adhesive to construct a laminated architecture. At the same time, ferrocene was employed as a catalyst to induce the in situ growth of carbon nanotubes (CNTs) under high-temperature conditions. This modification endowed the epoxy solvent-borne adhesive with not only high strength in atmospheric environments but also the capability to retain considerable mechanical performance at elevated temperatures. Experimental results demonstrated that when the graphene content in the epoxy solution fell within the range of 3.2–4%, the bonding strength exceeded 3 MPa within the temperature range of 1000–1300 °C. In particular, the adhesive exhibited excellent thermal shock resistance, with no degradation in strength observed after 15 thermal shock cycles at 1300 °C. Such exceptional performance was attributed to the formation of interlaminar CNTs generated after high-temperature treatment. Scanning electron microscopy (SEM) observations clearly revealed the laminated graphene sheets and in situ grown CNTs, confirming the feasibility of the strategy to enhance bonding efficacy by mimicking the nacre structure. This approach represented an innovative breakthrough for further research on the application of the “brick-and-mortar” structure in the bonding layer and the in situ growth of CNTs among lamellar graphene, while also providing detailed supporting data.

## 1. Introduction

Carbon fiber-reinforced carbon matrix (C/C) composites represent a paradigmatic class of lightweight, high-strength, and ultra-resistant structural materials, with extensive applications in aerospace engineering [1]. Nevertheless, their widespread adoption has concomitantly posed novel challenges, with the bonding of C/C materials emerging as a critical bottleneck [2,3]. High-temperature-resistant adhesive bonding, in particular, has proven to be an optimal solution for the assembly, fixation, sealing, and maintenance of C/C components [4]. Representative scenarios encompass the installation and securing of missile and rocket fairings, the fixation and thermal sealing of rocket engine nozzles, as well as the emergency repair of pits or cracks in spacecraft’s anti-aerothermal protection tiles or ablative tiles [5,6].

Adhesive bonding assembly has been extensively employed in aerospace engineering. Beyond enabling fundamental component joining, bonded assemblies also fulfill critical functions in sealing, thermal insulation, and vibration damping [7,8]. For instance, the B-58 heavy supersonic bomber employed adhesives to replace approximately 500,000 rivets, accounting for 85% of the total bonding area [9,10]. Likewise, a small aircraft utilizing adhesive bonding instead of riveting achieved a 20% weight reduction and a 30% strength enhancement [11]. However, the low surface polarity of C/C composites renders their bonding inherently challenging [12,13,14], which not only complicates the bonding effect under high-temperature conditions but also intensifies the demand for high-temperature adhesives with superior performance.

High-temperature-resistant adhesives suitable for carbon/carbon (C/C) composites are mainly classified into two categories: phosphate-based ceramic adhesives and resin-based ceramic precursor adhesives [15,16]. Benefiting from their rapid curing property at room temperature, phosphate-based ceramic adhesives exhibit significant advantages in emergency joining and repair scenarios of C/C components [17]. Although this type of adhesive can maintain bonding status over the whole temperature range, its bonding strength at room temperature is usually lower than 10 MPa, and the strength retention rate further decreases in high-temperature environments [18]. A typical case is the study by Wang et al., who reinforced aluminum phosphate adhesives by adding silicon carbide whiskers; even after heat treatment at 1300 °C, the bonding strength of the adhesive to C/C composites was still less than 5 MPa [19].

Resin-based ceramic precursor adhesives, by contrast, demonstrate higher mechanical performance, with their peak bonding strength exceeding 15 MPa [20,21]. The core mechanism of this type of material lies in the ceramization transformation that occurs at high temperatures. However, this process is accompanied by a volume shrinkage effect, which tends to cause interface failure at approximately 500 °C. Therefore, high-temperature pretreatment is generally required to ensure high strength of the bonded parts [22]. For instance, Wang et al. introduced in situ grown silicon carbide nanowires into a silicone resin matrix; after heat treatment at 1300 °C, the shear strength of the C/C joints was increased to 20.2 MPa [23].

The performance differences between the two types of adhesives stem from their intrinsic characteristics: phosphate-based materials possess a wide temperature range adaptability, but the brittleness of their inorganic phase network limits their strength; ceramic precursor materials achieve high strength through organic-inorganic transformation, yet volume changes during the pyrolysis process easily induce interface damage. A more fundamental issue is that a significant mismatch exists in the coefficient of thermal expansion (CTE) between the two types of adhesives and the C/C matrix (ΔCTE = 5–10 × 10^−6^ K^−1^). The thermal stress generated thereby will lead to the initiation of cracks in the bonded joints during thermal cycling, seriously restricting their service life [24,25]. Thus, the development of high-thermal-shock-resistant adhesives suitable for C/C composites holds significant importance.

Concurrently, the biomimetic nacreous structure, celebrated for its exceptional strength-toughness synergy, has gained prominence in materials science [26,27,28]. For instance, Ai et al. [29] fabricated nacre-inspired Ti6Al4V-(TiAlC/TiAl) laminated composites, revealing that the Ti2AlC/TiAl-TC4 composite with 20 vol.% Ti2AlC exhibited optimal flexural strength (564.86 MPa) and fracture toughness (39.15 MPa·m^1^/^2^). In addition, this bionic nacre-like structure also exhibits excellent thermal shock resistance. Li et al. [30] prepared Sm_0.5_Sr_0.5_CoO_3_-δ (SSC) ceramics with a nacre-like structure via the tape casting-solid-state reaction-laminating method. Comparative studies revealed that the nacre-like SSC ceramics possessed superior thermal shock resistance—attributed to the toughening effects of “SSC ‘brick’ pull-out” and crack deflection. Furthermore, under irradiation with high-concentration sunlight (21 W/cm^2^), the nacre-like SSC ceramics remained structurally intact and reached an equilibrium temperature of 870 °C, whereas the conventional bulk SSC ceramics cracked immediately upon exposure to high-concentration sunlight. In the field of polymers, graphene nanosheets have emerged as ideal reinforcement skeletons for constructing high-performance laminate structures, owing to their excellent chemical compatibility with polymeric matrices [31,32,33]. Li et al. [34] self-assembled a shell nacre-mimetic coating on the surface of carbon fibers. The coating is composed of carbon nanotube (CNT)-reinforced polyether amine (PEA)-polydopamine (PDA) and PDA-functionalized graphene oxide (GO@PDA). After optimizing the structure, the interfacial shear strength and fracture toughness of the composite material increased by 114.2% and 348.7%, respectively. This layered nacre-mimetic structure offers a novel approach to designing high-temperature-resistant adhesives with exceptional thermal shock resistance.

Inspired by the excellent mechanical properties (high strength and impact resistance) of the layered structure of nacre, this study proposed an innovative design strategy: the synergistic incorporation of ferrocene catalyst and graphene nanosheets into an epoxy solvent-borne adhesive system to construct a novel high-temperature-resistant adhesive with a layered reinforcing structure that performs well at both room temperature and high temperatures. At room temperature, graphene nanosheets, acting as a rigid reinforcing phase, are uniformly dispersed in the polymer matrix. They form strong interfacial bonding with resin segments through π-π conjugation, while the cross-linked network of the epoxy matrix ensures the adhesive’s firm adhesion to substrates. In a high-temperature environment, the polymer matrix undergoes thermo-oxidative degradation; meanwhile, the ferrocene catalyst decomposes into iron nanoparticles at high temperatures, which in situ catalyze the conversion of gaseous carbon sources into carbon nanotubes (CNTs) [35]. This realizes the three-dimensional lap connection and interconnection between CNTs and graphene nanosheets, forming a “graphene-CNT” layered reinforcing framework. This study provides valuable experimental data and theoretical insights for the development of high-performance heat-resistant adhesives, thereby advancing technological progress in aerospace engineering.

## 2. Materials and Methods

### 2.1. Materials

All raw materials were used as received without further purification. Graphene sheets (purity > 90%) and sodium dodecylbenzenesulfonate (analytical reagent, AR) were purchased from Shanghai Aladdin Biochemical Technology Co., Ltd., Shanghai, China. Isopropanol (AR) was supplied by Fuchen (Tianjin) Chemical Reagent Co., Ltd., Tianjin, China. Liquid E51 epoxy resin was obtained from Shanghai Aotun Chemical Technology Co., Ltd., Shanghai, China. Ferrocene (purity 99%) was provided by Shanghai Yien Chemical Technology Co., Ltd., Shanghai, China. Polyethylene glycol (number-average molecular weight, Mn = 2000) was purchased from Shanghai Maclin Biochemical Technology Co., Ltd., Shanghai, China.

### 2.2. Preparation Method

**Figure 1:**
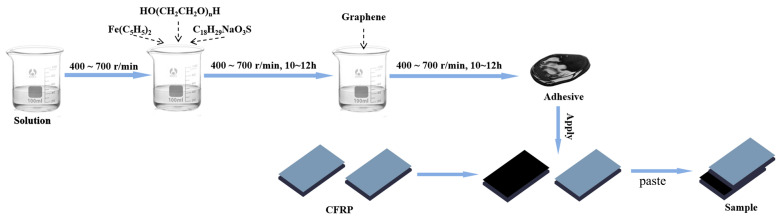
Steps of the experimental operation.

Drawing on the literature, graphene exhibits excellent chemical compatibility with resins. Accordingly, this study proposed a novel adhesive design to construct a biomimetic nacre-inspired structure, wherein the polymer served as the “mortar” and graphene as the “bricks.” Furthermore, the strategy aimed to enhance both the high-temperature bond strength retention and thermal shock resistance of joints by inducing in situ growth of carbon nanotubes (CNTs) at the interfaces between graphene sheets. The detailed experimental procedures are described as follows, with Figure 1 schematically illustrating the overall experimental workflow.

Isopropanol and epoxy resin were thoroughly mixed at room temperature and left to stand overnight. The resulting lower-layer slurry was used as the adhesive matrix. Under mechanical stirring at 400–700 r/min, polyethylene glycol (PEG, HO(CH_2_CH_2_O)_n_H, n = 2000), sodium dodecylbenzenesulfonate (SDBS, C_18_H_29_NaO_3_S), and ferrocene (Fe(C_5_H_5_)_2_) were added to the matrix in accordance with the proportions specified in Table 1. The mixture was stirred for 10–12 h until homogeneous. Subsequently, graphene sheets were incorporated and fully dispersed in the slurry via stirring at 400–700 r/min for an additional 10–12 h. The reaction vessel was kept sealed throughout the process to prevent volatilization-induced degradation of adhesive properties such as viscosity.

Carbon/carbon (C/C) composite specimens (40 mm × 10 mm × 5 mm) were first polished and ultrasonically cleaned with ethanol. No other surface treatments were performed. The treated specimens were then placed on a glass plate with the bonding surfaces facing upward. The adhesive coating process adhered to the principles of uniformity and defect-freeness, ensuring a smooth, bubble-free surface with a coated area of approximately 2 cm^2^. The brushing thickness of each adhesive layer was controlled at 200 μm using a coater. For bonded specimens with a single adhesive layer, the adhesive was applied to one of the bonding surfaces; after the adhesive became viscous, bonding was performed, during which a certain amount of adhesive was squeezed out. For specimens with three adhesive layers, the adhesive was first applied to both bonding surfaces; after drying, an additional layer of adhesive was brushed on before bonding. The operation procedure for the five adhesive layers was similar. Due to the different graphene contents in various adhesives, there was a significant impact on the overall thickness. After the adhesive was cured, the thickness of the three-layer G4 adhesive was approximately 90 μm, while that of the three-layer G6 adhesive was about 150 μm. Owing to workload constraints, only the brushing thickness was controlled in this study. The assembled samples were immobilized and cured at room temperature before being calcined.

### 2.3. Testing and Characterization

High-temperature calcination was conducted using a tubular furnace filled with argon gas, where the sample was calcined for one hour at a given temperature. Following thermal treatment at different temperatures, the room-temperature shear strength tests were conducted using a universal testing machine (UTM). Each group of tests consisted of five samples, and the final result was calculated as the average value of these samples. Different adhesives were tested using a Rigaku X-ray diffractor (model: D/Max2500v/PC, Tokyo, Janpan) and Thermo Fisher Scientific fourier transform infrared diffractometer (model: iS50, Waltham, MA, USA) instruments for composition analysis, molecular structure characterization, and chemical composition determination. The adhesive samples were also tested using a scanning electron microscope (SEM, model: Nanosem 430, FEI, Philadelphia, PA, USA) and a transmission electron microscope (TEM, model: Tecnai G2 F20; FEI, Philadelphia, PA, USA). In addition, the viscosity of the adhesive slurry was measured at varying shear rates using a rotational viscometer. Before each test, the rotor was thoroughly cleaned, and a fresh viscosity calibration was performed. The contact angle was determined at ambient temperature using a contact angle goniometer. Each sample was tested five times in repetition, after which the average values and errors of viscosity and contact angle were calculated.

## 3. Results and Discussion

### 3.1. Viscosity and Wettability

Viscosity stands as a key performance parameter of adhesives, governing their coating processability, flowability, and rheological consistency. Notably, while higher viscosity often correlates with enhanced cohesive strength of the adhesive matrix, it must be balanced against spreading behavior. For effective bonding, the adhesive must exhibit adequate wetting and spreading capacity on the substrate surface, ensuring the formation of a thin, homogeneous coating free from defects, while facilitating intermolecular interactions between adhesive molecules and substrate surface groups. Furthermore, the thermodynamic driving force behind wetting (characterized by the contact angle) directly influences the formation of intimate interfacial contact, which is a prerequisite for achieving robust adhesion. Optimizing viscosity to match the substrate’s topography enables sufficient penetration into surface micro-asperities, thereby maximizing van der Waals forces and chemical bonding at the interface.

**Figure 2:**
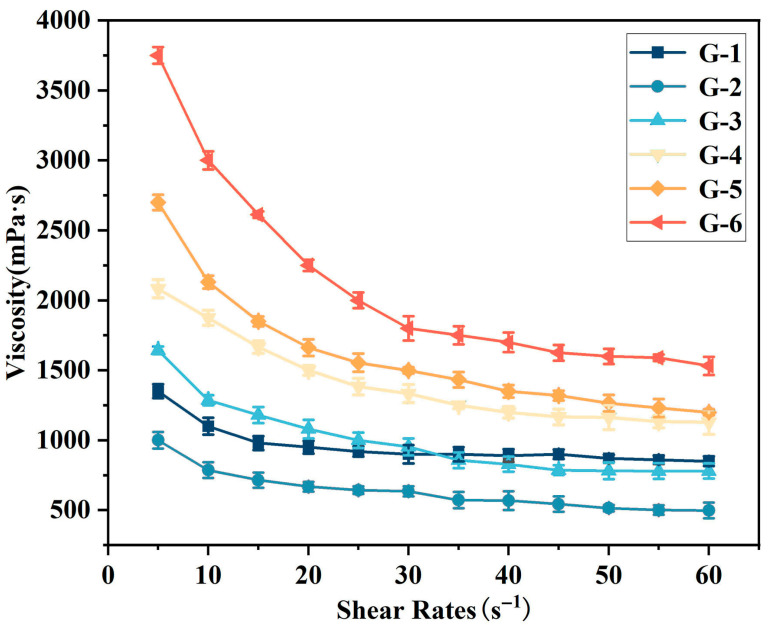
The variation in viscosity of different G-X epoxy solvent-borne adhesives with shear rate.

Firstly, the dependence of viscosity on shear rate was investigated. As illustrated in Figure 2, the viscosity of G-X epoxy solvent-borne adhesives varies with shear rate: specifically, viscosity decreases progressively with increasing shear rate, exhibiting shear-thinning behavior. Additionally, the viscosity of G-X adhesive is closely related to the amount of graphene added, with higher content resulting in higher viscosity. Graphene sheets and adhesive matrix molecules (e.g., resin segments) form strong interactions through van der Waals forces, hydrogen bonds, and other mechanisms, which significantly impede the free movement of matrix molecules. Meanwhile, the sheets tend to agglomerate due to intermolecular forces; the resulting agglomerates further increase the steric hindrance within the system, leading to a sharp rise in internal frictional resistance when the adhesive flows—ultimately manifesting as increased viscosity. At a shear rate of 5 s^−1^, the G-6 adhesive exhibits the highest viscosity (3750 mPa·s), while the G-1 adhesive shows the lowest (1000 mPa·s). Similarly, at 60 s^−1^, G-6 retains the highest viscosity (1700 mPa·s), with G-1 remaining the lowest at 500 mPa·s.

**Figure 3:**
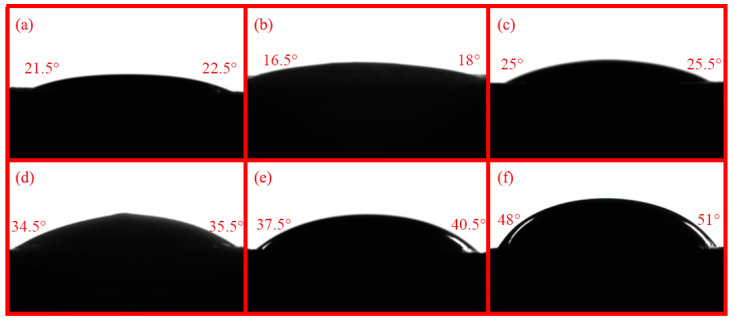
The typical wetting behavior of different G-X epoxy solvent-borne adhesives on the surface of C/C composites: (**a**) G-1, (**b**) G-2, (**c**) G-3, (**d**) G-4, (**e**) G-5, (**f**) G-6.

**Figure 4:**
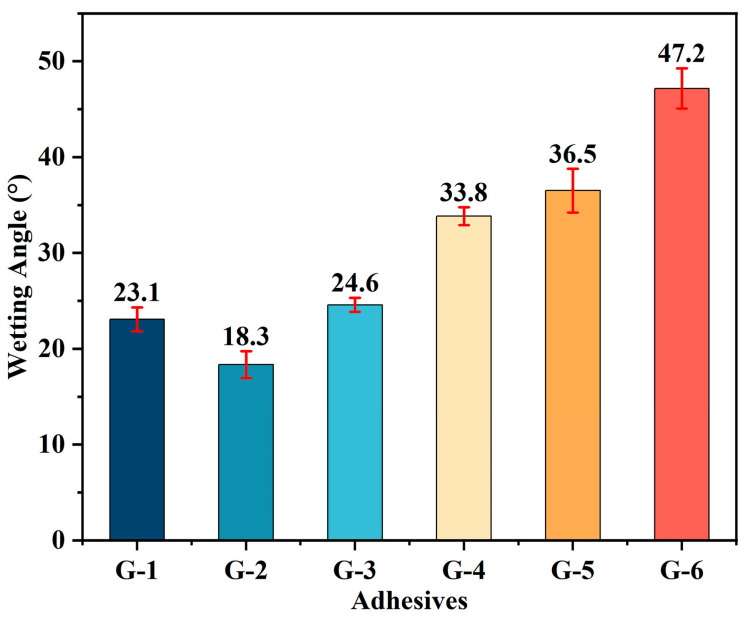
Contact angles of different G-X epoxy solvent-borne adhesives on the surface of C/C composites.

The wetting behavior of G-X adhesives on the surface of C/C composites is shown in Figure 3. The contact angles of G-X adhesive solutions are presented in Figure 4, which includes duplicate measurements and calculated average values for each formulation. These results confirm that all epoxy solvent-borne adhesives demonstrate favorable wetting on the carbon composite surface, with G-2 exhibiting the most superior wetting performance due to its extremely low contact angles. As the amount of graphene added increases, the viscosity of the G-X adhesive increases, while its wettability decreases. The elevated viscosity directly reduces the fluidity and spreading ability of the adhesive on the surface of the adherend substrate. Additionally, the introduction of graphene sheets alters the surface tension of the adhesive system: their relatively hydrophobic surface properties may cause the adhesive’s surface tension to deviate from the range compatible with the substrate. At the same time, agglomerated sheets form a microscopically rough structure at the adhesive-substrate interface, reducing the effective contact area. These two effects collectively prevent the adhesive from thoroughly wetting the substrate, ultimately resulting in decreased wettability. Among the tested adhesives, G-6 exhibits the largest average contact angle, at 47.2°, while G-1 shows the smallest average contact angle, at 18.3°. Despite exhibiting differences, all these adhesives demonstrate wettability on the C/C composite surface.

### 3.2. Bonding Performance

Bonding strength, specifically shear strength in this study, refers to the shear force sustained at the bonded interface over a defined period, which directly dictates the practical applicability of the adhesive. Diverse application scenarios necessitate distinct shear strength requirements, and the multiple influencing factors governing this property are explored in subsequent sections.

#### 3.2.1. Effect of Coating Layer Number on Bonding Performance

The number of coating layers significantly influences the bonding performance of adhesives, as varying layer counts can alter the thickness of the adhesive film, interfacial contact area, and stress distribution at the bonded interface. This section investigates explicitly how varying the number of coating layers affects the shear strength of bonded joints, aiming to identify the optimal layer count for enhanced bonding performance. To determine the optimal number of adhesive coatings, we selected the G-1 adhesive with one, three, and five layers, and heated it to 250 °C and 1300 °C for one hour in an argon protective atmosphere.

**Figure 5:**
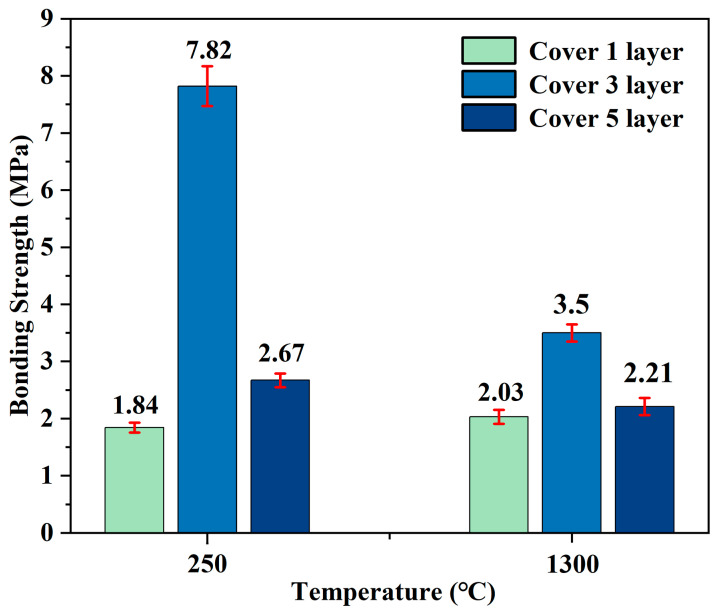
Bonding strength of G-1 adhesive with different layer numbers after treatment at 250 °C and 1300 °C.

As depicted in Figure 5, for the G-1 adhesive cured at 250 °C, the bonding strength is the highest (7.82 MPa) when there are three coating layers, while it is the lowest (1.84 MPa) with one coating layer. At 1300 °C, the three-layer coating yields a bonding strength of 3.50 MPa. When the number of layers increases to five, the shear strength shows a downward trend compared to three layers.

These data indicate that with the increase in the number of layers to three, the shear strength reaches the maximum value, which may be because three layers of coating can form a moderate thickness of adhesive film, ensuring sufficient interfacial contact and uniform stress distribution. However, when the number of layers increases to five, the shear strength decreases. This may be because excessive coating layers result in a too-thick adhesive film, which is prone to generating internal defects during curing or calcination, thereby reducing bonding performance. Therefore, regardless of whether the curing process is carried out at 250 °C or the calcination at 1300 °C, three coating layers are optimal. These results further confirm that the number of coating layers has a crucial impact on the bonding performance of the adhesive.

**Figure 6:**
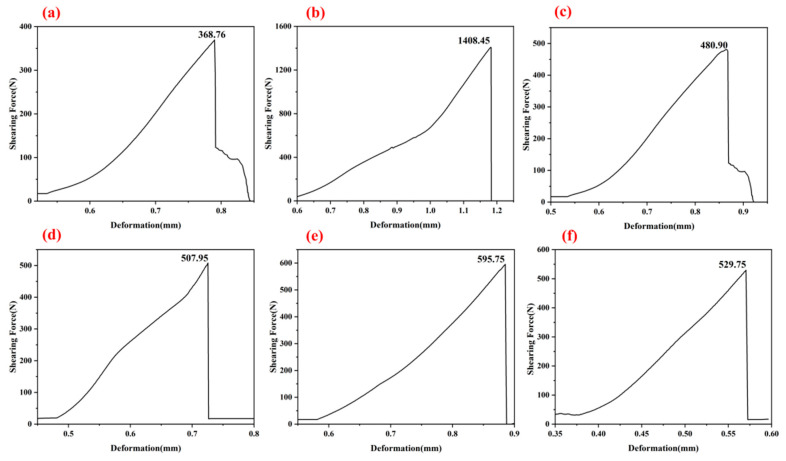
The typical shearing force–deformation curves corresponding to Figure 5 ((**a**): 250 °C, 1 layer; (**b**): 250 °C, 3 layers; (**c**): 250 °C, 5 layers; (**d**): 1300 °C, 1 layer; (**e**): 1300 °C, 3 layers; (**f**): 1300 °C, 5 layers).

Figure 6 illustrates the shearing force–displacement curves for the G-1 adhesive with one, three, and five coating layers after thermal treatment at 250 °C and 1300 °C (as shown in Figure 6a–f). All curves exhibit an overall ascending trend, with the three-layer G-1 adhesive displaying a steeper slope and higher stiffness across both temperature conditions. These curves all indicate that the fracture of the bonded component is brittle fracture. In this part of the experiment, we determined that the three-layer adhesive is the optimal bonding method. In subsequent experiments, we uniformly adopted the three-layer bonding structure and investigated in detail the bonding effects of different adhesives after treatment at different temperatures.

#### 3.2.2. Effect of Temperature on Bonding Performance

Under the condition of three coating layers, the bonding strength of different adhesives after curing at 250 °C, followed by treatment at elevated temperatures, was investigated. All bonded specimens were dried at 80 °C before being cured at 250 °C. This experimental setup enables a systematic evaluation of how elevated temperature treatments (beyond the initial curing stage) affect the bonding performance of various adhesives, with a consistent three-layer configuration ensuring comparability across different temperature conditions.

**Figure 7:**
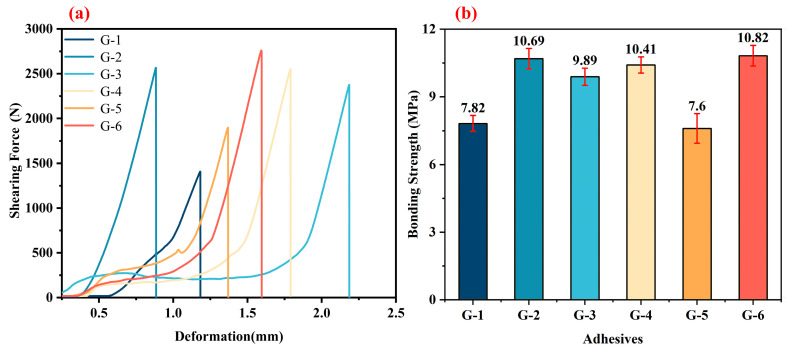
The typical shearing force–deformation curves (**a**) and the corresponding shear strengths (**b**) for the bonded joints with different adhesives after treatment at 250 °C.

Figure 7a presents the shear force–deformation curves of bonded joints using different adhesives (three-layer configuration) after treatment at 250 °C. The curves exhibit a similarity, with a linear correlation between shear force and deformation, indicating that the bonding layer is rigid and the fracture mode is brittle. The continuous curve indicates a uniform distribution of the bonding layer structure. As shown in Figure 7b, at 250 °C, the G-2 and G-6 adhesives achieve the highest bonding strengths, reaching 10.69 MPa and 10.82 MPa, respectively. In contrast, the G-5 adhesive exhibits the lowest bonding strength of 7.6 MPa.

**Figure 8:**
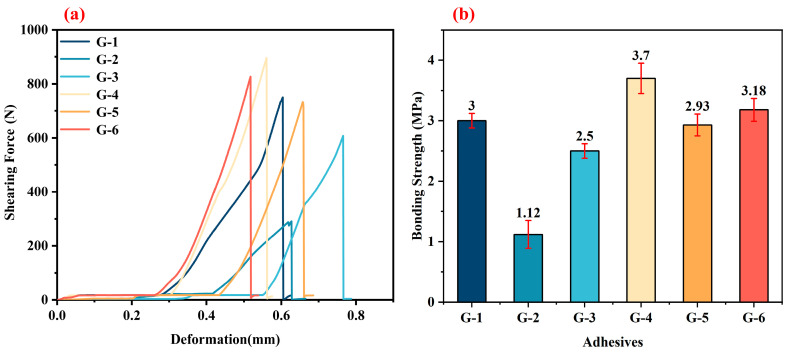
The typical shearing force–deformation curves (**a**) and the corresponding shear strengths (**b**) of the bonded joints with different adhesives after treatment at 1000 °C.

Figure 8 illustrates the bonding strength of G-X alcohol-based adhesives after treatment at 1000 °C. The shear force–deformation curves of the six adhesives are similar, without significant fluctuations. This indicates that the structure of the G-X adhesive layer remains a good structural distribution at 1000 °C, with no apparent defects. After the 1000 °C treatment, G-4 exhibits the optimal bonding strength (3.7 MPa), G-2 shows the poorest (1.12 MPa), while the bonding strengths of G-1 and G-6 are both approximately 3 MPa. Compared with the strength after treatment at 250 °C, the strength after treatment at 1000 °C decreases significantly, which is attributed to the decomposition of the epoxy resin. However, the ability to still provide a specific bonding strength at 1000 °C stems from the formation of a structure where carbon nanotubes (CNTs) connect stacked graphene layers (details below).

**Figure 9:**
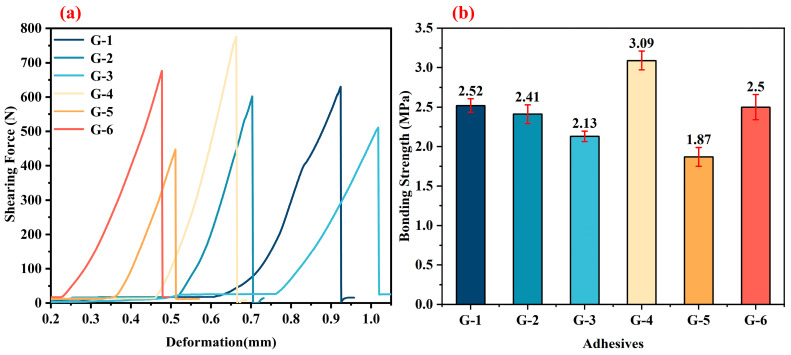
The typical shearing force–deformation curves (**a**) and the corresponding shear strengths (**b**) of the bonded joints with different adhesives after treatment at 1500 °C.

Figure 9 presents the mechanical performance characterization of bonded joints using different G-X epoxy solvent-borne adhesives (three-layer configuration) after treatment at 1500 °C. Figure 9a shows the shearing force–deformation curves. It can be observed that the curves of various adhesives (G-1 to G-6) exhibit certain regularity. With the increase in deformation, the shearing force generally shows a rising trend, and then drops rapidly after reaching its peak value, which is a typical mechanical response characteristic of the bonding failure process. The similarity of the shearing force–deformation curves in Figure 9a also indicates that the internal defect levels of different adhesive layers after 1500 °C treatment are relatively close, and the overall bonding failure modes are similar. Figure 9b is the statistical histogram of bonding strength. After treatment at 1500 °C, G-4 shows the optimal bonding strength, reaching 3.09 MPa. The bonding strength of G-5 is the lowest, only 1.87 MPa. The bonding strengths of G-1, G-2, G-3, and G-6 are 2.52 MPa, 2.41 MPa, 2.13 MPa, and 2.5 MPa, respectively. This set of data reflects the influence of high-temperature treatment at 1500 °C on the bonding performance of different G-X epoxy solvent-borne adhesives and also provides a basis for screening adhesives suitable for high-temperature service environments.

Under the condition of three coating layers, the bonding performance of different adhesives varies significantly with temperature treatments. After treatment at 250 °C, G-6 exhibits the highest bonding strengths (10.82 MPa) with good structural uniformity (linear correlation of shear force–deformation curves). At 1000 °C, the bonding strengths of all adhesives decrease significantly due to epoxy resin decomposition, yet G-4 still achieves the highest strength (3.7 MPa), with the maintenance of specific bonding strength attributed to the formation of carbon nanotube-connected stacked graphene structures. After treatment at 1500 °C, G-4 demonstrates the optimal bonding strength (3.09 MPa), while G1 and G6 also exhibit relatively high bonding strengths (2.52 MPa and 2.5 MPa, respectively). Taken together, G4 and G6 exhibit the best bonding performance. Therefore, this work focuses on G4 and G6 adhesives as the key research objects, and further investigates their bonding strengths after treatment at different temperatures within the range from the curing temperature to 1500 °C.

**Figure 10:**
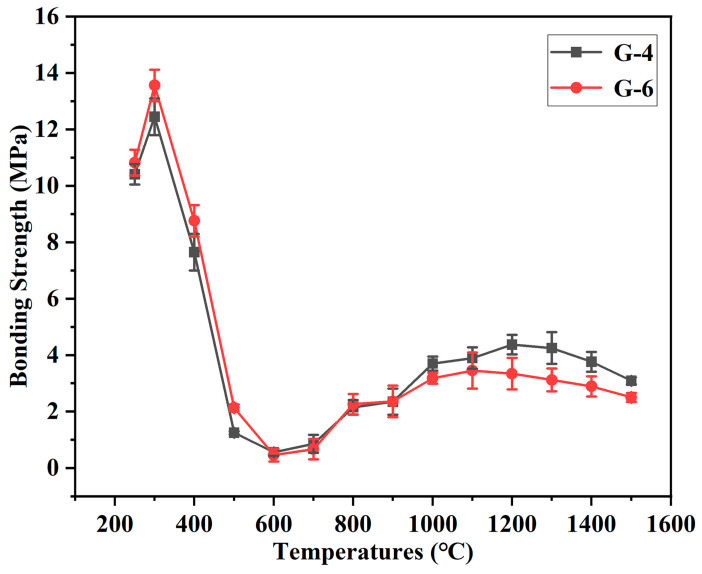
The bonding strength of G-4 and G-6 adhesives after treatment at different temperatures.

To clarify the bonding performance of this type of adhesive after treatment across the whole temperature range from 250 °C to 1500 °C, the bonding effects of G-4 and G-6 adhesives are tested following treatment at different temperatures, as shown in Figure 10. The two adhesives exhibit a consistent trend in bonding strength evolution with temperature, and the difference in their strengths is relatively small. Owing to the relative stability of the epoxy resin, the adhesives maintain high bonding strength when the treatment temperature is not higher than 400 °C. Although 400 °C causes a certain degree of thermal decomposition of the epoxy resin, the bonding strength can still be maintained at approximately 8 MPa. After treatment at 500 °C, the epoxy resin decomposes severely, and the strength decreases to 1–2 MPa at this point. The decomposition is even more severe at 600 °C, with the strength being less than 1 MPa at this temperature. Since 700 °C is not sufficient to catalyze the formation of CNTs, the bonding strength remains low after treatment at 700 °C. When the temperature reaches 800 °C, the strength increases to 2 MPa; this is precisely because the formation of CNTs between graphene sheets promotes the generation of an interlayer lap structure. As the treatment temperature continues to rise, the bonding strength of both adhesives increases. After treatment at 1200 °C and 1300 °C, the strength of G-4 adhesive exceeds 4 MPa.

Compared with existing advanced engineering high-temperature-resistant adhesives [23,24,36], the adhesive developed in this work actually exhibits lower bonding strength. However, this biomimetic layered junction structure exhibits the following three advantages after high-temperature treatment: firstly, it is an all-carbon structure, which makes the adhesive layer consistent with the C/C material composition; The second is that the coefficient of thermal expansion is consistent, which makes the bonded parts have better thermal shock resistance; Thirdly, the connecting parts have good thermal and electrical conductivity, which gives the adhesive a certain degree of functionality. In summary, this new type of adhesive is suitable for connecting non-load-bearing components of C/C, particularly for components that require rapid heat dissipation.

### 3.3. Thermal Shock Resistance

**Figure 11:**
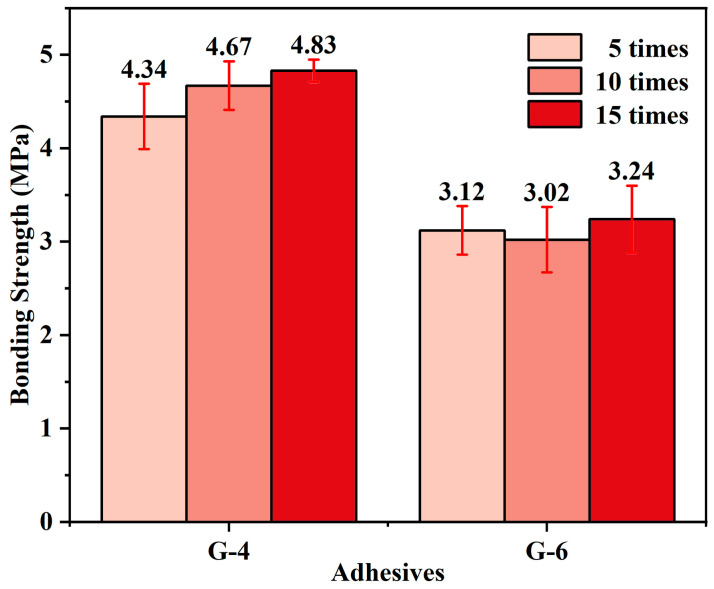
The bonding strength of G4 and G6 adhesives after different thermal shocks at 1300 °C.

Thermal shock resistance is crucial for high-temperature adhesives. G-4 and G-6 adhesives were dried at 80 °C after primary coating, cured at 250 °C after bonding, and calcined at 1300 °C for 5, 10, and 15 thermal shocks. Figure 11 presents the variation in bonding strength of G-4 and G-6 adhesives with the number of thermal shocks. It is evident from the data that after 15 thermal shocks, G-4 exhibits the highest bonding strength (4.83 MPa), while G-6 shows a lower bonding strength (3.24 MPa). A distinct trend in bonding strength evolution is observed between the two adhesives: the bonding strength of G-4 increases gradually with the increase in thermal shocks, indicating its excellent stability and adaptability under repeated high-temperature calcination. In contrast, the strength of G-6 remains almost unchanged with an increase in thermal shock frequency, maintaining a value of around 3.1 MPa. The thermal shock tests of these two adhesives both demonstrate that the adhesive developed in this work has excellent thermal shock resistance.

**Figure 12:**
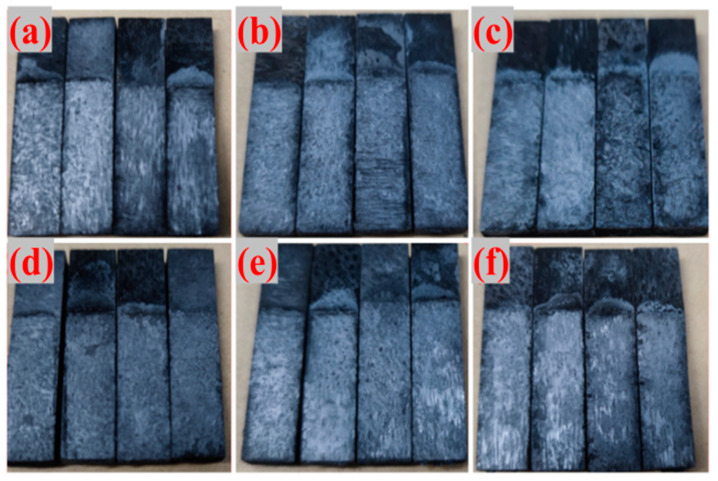
The fracture surfaces of epoxy solvent-borne adhesives G-4 and G-6 after thermal shocking ((**a**) G-4, 5 times; (**b**) G-4, 10 times; (**c**) G-4, 15 times; (**d**) G-6, 5 times; (**e**) G-6, 10 times; (**f**) G-6, 15 times).

To further explore the underlying mechanism behind the differing thermal shock resistances, Figure 12 displays the adhesive fracture surfaces of G-4 and G-6 after 5, 10, and 15 thermal shocks. As the number of thermal shocks increases, the fracture surface of the adhesive layer undergoes a noticeable transformation: it becomes gradually denser and flatter. This morphological change suggests that the internal structure of the adhesive layer is optimized during repeated high-temperature treatments, with reduced defects and improved interfacial bonding, which may contribute to the gradual increase in its bonding strength.

### 3.4. XRD Analysis

**Figure 13:**
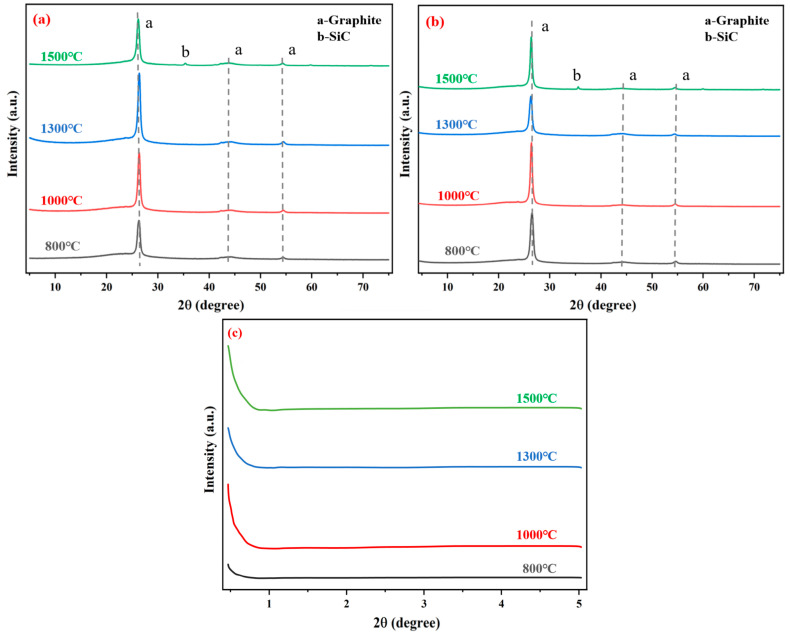
(**a**) XRD diffraction pattern of G-4 adhesive after treatment at different temperatures, (**b**) XRD diffraction patterns of G-6 adhesive after treatment at different temperatures, (**c**) small diffraction angle XRD patterns of G-4 after treatment at different temperatures.

Material properties are inherently correlated with microstructure and chemical composition, both of which undergo significant alterations as temperature changes. Figure 13 displays the XRD diffraction profiles of G-4 and G-6 adhesives. The prominent diffraction peaks in the profiles correspond to the adhesive-graphene layered skeleton and carbon phases. Specifically, in addition to the characteristic peaks of graphene, the presence of peak “a” further confirms the contribution of carbon components in the adhesive system. These pure carbon components, with their excellent thermal conductivity and chemical stability, play a crucial role in enhancing the bonding performance. They can fill the micro-voids at the bonding interface, improve the interfacial contact area, and form a continuous conductive network, thereby promoting the transfer of stress and heat at the interface and enhancing the overall bonding strength.

Notably, a small diffraction peak “b” emerges in the XRD patterns of adhesives treated at 1500 °C. By comparing it with standard reference cards, this peak is identified as corresponding to SiC. It is thus speculated that when the temperature reaches 1500 °C, a minor reaction takes place between a small amount of carbon in the adhesive and silicon from the ceramic container, leading to the formation of SiC. The generation of SiC, which has high hardness and good high-temperature stability, may have a specific reinforcing effect on the adhesive layer; however, due to the small amount, its impact on the overall bonding performance may be limited.

In addition, Figure 13a,b show an upward curve at small diffraction angles. To explore potential omissions in phase identification, XRD small-angle diffraction analysis was performed on the G-4 sample. This analysis revealed a diffraction peak with a 2θ value less than 1°, which is attributed to the mesopore effect. The mesopore structure in the adhesive layer can act as a buffer zone during the bonding process. When the adhesive is subjected to external forces or temperature changes, the mesopores can absorb and disperse the stress, reducing the probability of crack initiation and propagation. Moreover, the mesopore structure can also provide channels for the diffusion of small molecules, which is beneficial for the curing reaction of the adhesive and the improvement of bonding performance. However, an excessive number of mesopores may lead to a decrease in the density of the adhesive layer, thereby reducing the bonding strength. Therefore, the appropriate mesopore structure is of great significance for optimizing the bonding performance of the adhesive.

### 3.5. FTIR Analysis

To further characterize the molecular components of the adhesives after high-temperature treatment and complement the XRD-based structural analysis, infrared (IR) spectroscopy was performed. The IR test helps identify residual chemical bonds and functional groups, which are critical for linking microstructural features (e.g., carbon phases, graphene layers identified via XRD) to post-high-temperature bonding performance.

**Figure 14:**
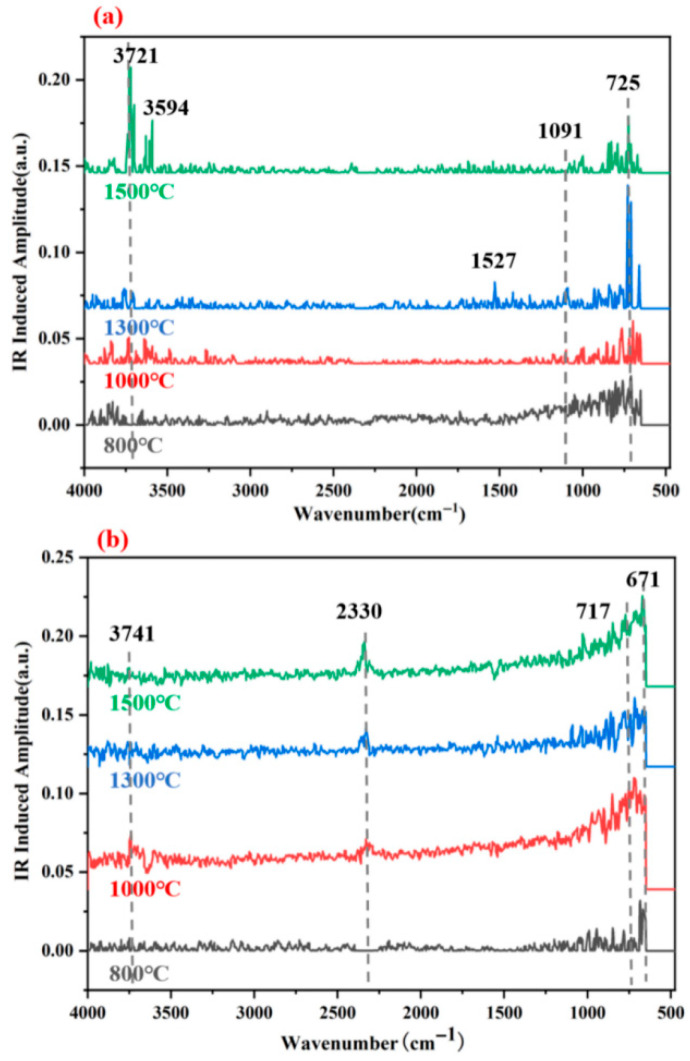
FTIR patterns of G-4 (**a**) and G-6 (**b**) adhesives after treatment at different temperatures.

Figure 14 presents the infrared spectra of G-4 and G-6 adhesives after treatment at different temperatures. Notably, organic functional groups (e.g., most C-H, C=O in aliphatic or unstable aromatic systems) typically decompose or diminish significantly above 1000 °C. In line with this, the spectra exhibit minimal intense peaks associated with labile organic moieties. In Figure 14a (G-4), a set of weak absorption peaks near 725 cm^−1^ and in Figure 14b (G-6) within 671–750 cm^−1^ are attributed to residual aromatic C=C stretching and C-H bending vibrations—likely from thermally stable benzene ring structures (e.g., graphitized domains), and consistent with the carbon-rich framework observed in XRD.

A faint absorption peak near 1091 cm^−1^ in both spectra, assigned to C-O stretching vibrations [35], may originate from thermally stable ether linkages or residual inorganic-organic hybrid species, as bulk organic carbonyls would have decomposed at high temperatures. In Figure 14b (G-6), the weak peak near 2330 cm^−1^, tentatively assigned to C-H stretching in ferrocene derivatives, likely reflects residual thermally stable organometallic moieties (ferrocene derivatives are more heat-resistant than typical organics) that survived 1000 °C treatment but diminished in intensity. The broad absorption in the 3500–3750 cm^−1^ region is attributed to residual O-H (from adsorbed water or stable hydroxyl groups on carbon surfaces) rather than labile N-H or organic O-H, as these would have decomposed—consistent with XRD-identified mesopores that may trap trace water.

Overall, the spectra indicate that most organic components decomposed at 1000 °C, with residual peaks corresponding to thermally stable aromatic or organometallic species, reinforcing the XRD finding that carbon-based frameworks (not labile organics) dominate post-high-temperature bonding performance.

### 3.6. Microstructure Analysis

**Figure 15:**
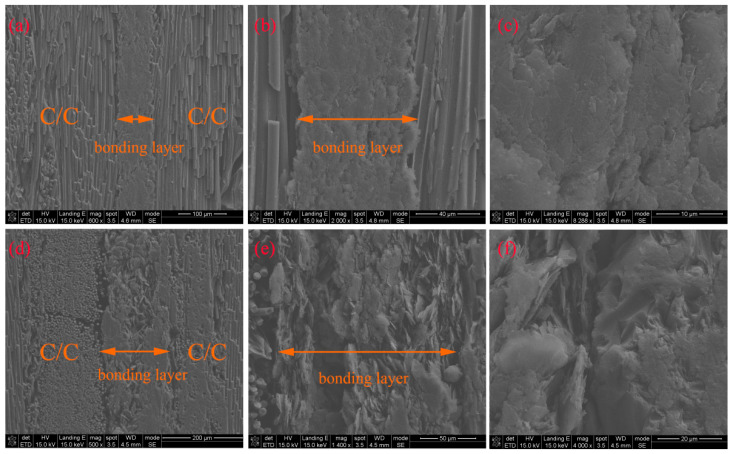
Cross-sectional SEM images of C/C joints bonded by G-4 (**a**–**c**) and G-6 (**d**–**f**) adhesives after treatment at 1500 °C.

Figure 15 shows the cross-sectional SEM images of C/C joints bonded with G-4 and G-6 adhesives after treatment at 1500 °C. The bonding layer corresponding to G-4 is very dense with a thickness of approximately 90 μm, where graphene is barely visible, and carbon nanotubes are even harder to detect. In contrast, the bonding layer of G-6 is relatively loose, with a thickness of about 150 μm, and partial lamellar graphene is exposed, which is why the strength of G-6 is lower than that of G-4. Carbon nanotubes (CNTs) are hardly observable in Figure 15; therefore, it is necessary to observe the fracture surfaces.

To verify the reinforcement effect of carbon nanotubes (CNTs) grown in situ on graphene on the bonding strength of adhesives under high-temperature conditions, the morphologies of fracture surfaces and adhesive layer surfaces of different adhesives were observed. It is scientifically plausible that graphene can serve as a substrate for in situ CNTs growth at high temperatures, especially with ferrocene acting as a catalyst—this aligns with the catalytic mechanism of CNTs growth, where graphene provides nucleation sites and high temperatures facilitate the decomposition of precursors to form CNTs.

**Figure 16:**
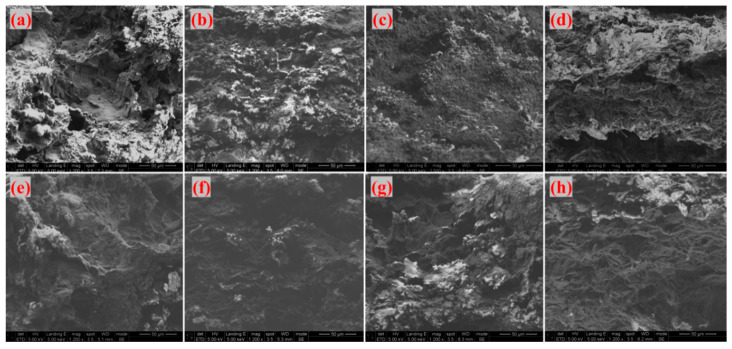
SEM images of the fracture surfaces of different adhesives ((**a**) G-4-800 °C; (**b**) G-4-1000 °C; (**c**) G-4-1300 °C; (**d**) G-4-1500 °C; (**e**) G-6-800 °C; (**f**) G-6-1000 °C; (**g**) G-6-1300 °C; (**h**) G-6-1500 °C).

As shown in Figure 16, which presents SEM images of the fracture surfaces of different adhesives of G-4 and G-6 after treatment at different temperatures. the fracture surfaces in Figure 16a (G-4 at 800 °C) and (e) (G-6 at 800 °C) exhibit significant fluctuations, with obvious sheet-like graphene visible. At this temperature, the thermal energy is insufficient to trigger extensive CNT growth, so the fracture surface is dominated by graphene morphology. With increasing temperature, the fracture surfaces in Figure 16b (G-4 at 1000 °C), (c) (G-4 at 1300 °C), (f) (G-6 at 1000 °C), and (g) (G-6 at 1300 °C) show reduced fluctuation, accompanied by the appearance of granular fine protrusions. Combined with the aforementioned infrared spectroscopy (which indicated residual thermally stable species related to carbon structures) and XRD analysis (confirming carbon-based frameworks), these protrusions are preliminarily inferred to be traces of CNT growth—consistent with the elevated temperature promoting precursor decomposition and CNT nucleation/growth on graphene. When the temperature reaches 1500 °C, as shown in Figure 16d (G-4 at 1500 °C) and (h) (G-6 at 1500 °C), the fracture surfaces exhibit a folded morphology. This is attributed to the exposure of sheet-like graphene and the disappearance of punctate protrusions, which is scientifically reasonable: excessively high temperatures may cause CNTs to undergo graphitization or structural damage, or even lead to the decomposition of catalyst components, thereby inhibiting further CNT growth.

**Figure 17:**
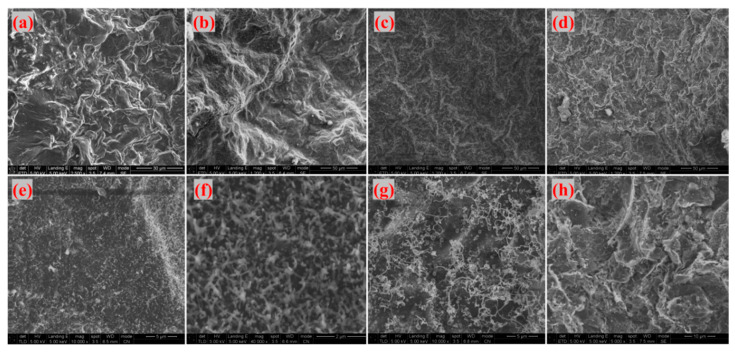
Microtopography of G-4 adhesive after treatment at different temperatures ((**a**,**e**) 800 °C; (**b**,**f**) 1000 °C; (**c**,**g**) 1300 °C; (**d**,**h**) 1500 °C).

**Figure 18:**
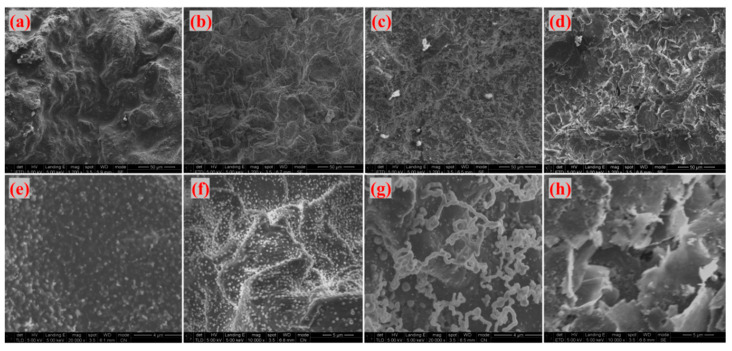
Microtopography of G-6 adhesive after treatment at different temperatures ((**a**,**e**) 800 °C; (**b**,**f**) 1000 °C; (**c**,**g**) 1300 °C; (**d**,**h**) 1500 °C).

Figure 17 and Figure 18 display SEM images of G-4 and G-6 adhesives treated at different temperatures, respectively. At 800 °C, 1000 °C, and 1300 °C, the adhesive sample surfaces remain relatively intact. Under high magnification, a large number of linear structures—identified as catalytically generated CNTs—are observed, with the densest distribution at 1300 °C. This indicates that 1300 °C is the optimal temperature for CNT growth: at 800 °C, the temperature is too low to fully activate the catalyst and precursor decomposition; at 1000 °C, CNT growth initiates but is not yet optimal; 1300 °C, meanwhile, provides sufficient thermal energy to promote extensive CNT growth without causing structural damage. When the temperature reaches 1500 °C, CNTs disappear, the sample surfaces become fragmented, and laminated sheet-like graphene is exposed—this further confirms that ultra-high temperatures disrupt the CNT structure, as excessive thermal energy can break the C-C bonds in CNTs or cause catalyst evaporation, making it impossible for CNTs to maintain their reinforcing effect on the adhesive structure.

**Figure 19:**
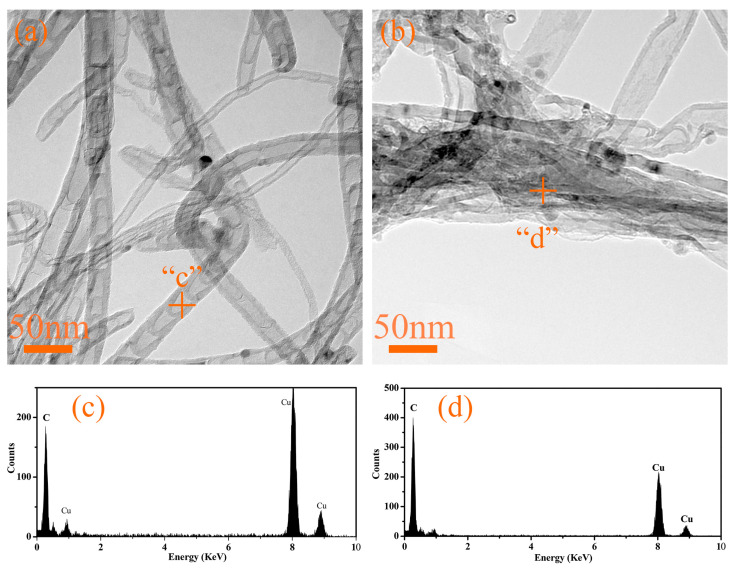
TEM images of the in situ nanotubes in G4 (**a**,**c**) and G6 (**b**,**d**) adhesives after treatment at 1300 °C.

To further confirm that the nanotubes are carbon nanotubes (CNTs), we conducted transmission electron microscopy (TEM) characterization on G4 and G6 samples after heat treatment at 1300 °C, with the results presented in Figure 19. As shown in Figure 19a,b, the observed structures exhibit the typical morphological features of carbon nanotubes. Specifically, the CNTs formed in G4 have a diameter ranging from approximately 10 to 20 nm, showing excellent dispersion with minimal agglomeration. In contrast, individual CNTs in G6 can reach a maximum diameter of up to 40 nm, and they tend to agglomerate significantly, forming interconnected aggregates with a dimension exceeding 100 nm. Energy-dispersive X-ray spectroscopy (EDS) analysis revealed that both CNT samples are composed exclusively of carbon. No iron element was detected, and the presence of copper is attributed to the copper grids used for sample support.

The extensive presence of CNTs in the SEM images confirms the validity of the experimental design: this study enhances bonding strength by utilizing ferrocene-catalyzed CNT growth on graphene. This mechanism is supported by the correlation between CNT density and bonding strength—higher CNT density at 1300 °C corresponds to better bonding performance, while CNT disappearance at 1500 °C aligns with strength degradation. These findings provide valuable insights for subsequent research on graphene-reinforced resin-based adhesives, particularly regarding the optimization of high-temperature treatment processes to regulate CNT growth and maximize reinforcing effects.

### 3.7. Bonding Mechanism

**Figure 20:**
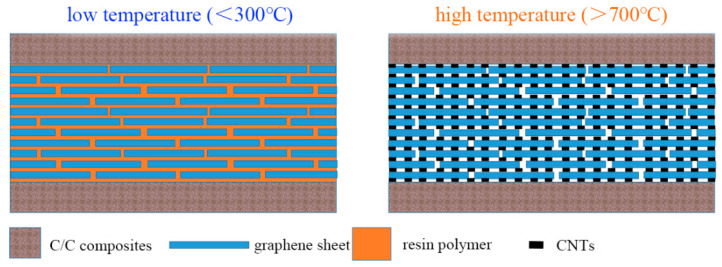
Schematic diagram of adhesive layer at low and high temperatures.

Drawing inspiration from the bionic nacre structure, graphene is incorporated into the adhesive to construct a laminated structure, while ferrocene is utilized to catalyze the in situ growth of carbon nanotubes (CNTs) at high temperatures. This design enables the resin (epoxy)-based adhesive to not only achieve high strength in atmospheric environments but also retain a certain level of bonding performance under high-temperature conditions (argon atmosphere). Figure 20 presents a schematic diagram of the heat-resistant adhesive with a laminated structure, intuitively illustrating the core design concept of the adhesive layer.

At temperatures below 300 °C, the adhesive exhibits a “brick-and-mortar structure”: graphene sheets act as the “bricks”, providing a skeletal support and contributing fundamental strength to the overall structure by virtue of their excellent mechanical properties; epoxy resin serves as the “mortar”, filling the gaps between graphene sheets. Through the curing reaction, the resin forms a continuous matrix phase, tightly bonding the graphene sheets together and significantly enhancing the bonding effect after curing. At this stage, the synergistic effect between graphene and resin not only leverages the high-strength characteristic of graphene but also utilizes the good wettability and adhesiveness of the resin, enabling the adhesive to stably transfer stress in ambient and medium-to-low temperature environments and exhibit high bonding strength.

When the temperature rises above 700–800 °C, the epoxy resin gradually decomposes under high temperature. At the same time, ferrocene exerts a catalytic effect in this temperature range, promoting the in situ growth of CNTs from carbon sources in the decomposition products on the surface of graphene and in the gaps between sheets. These CNTs act as a new “mortar”, interconnecting the originally relatively independent graphene sheets to form a reticulated mesoporous structure, as shown in Figure 20. In this high-temperature-formed structure, graphene still functions as the “bricks”, maintaining the fundamental skeleton and mechanical support of the structure; CNTs serve as the “mortar”, not only filling the gaps between graphene sheets but also firmly binding the graphene sheets through three-dimensional reticulated connections, constructing a stable spatial structure. This new “brick-and-mortar structure” with graphene as bricks and CNTs as mortar, relying on the excellent high-temperature resistance of carbon materials, can withstand high temperatures up to 1500 °C.

## 4. Conclusions

This work addressed the bonding needs of carbon/carbon (C/C) composites in extreme high-temperature environments. Inspired by the bionic nacre “brick-mortar” structure, a graphene-reinforced epoxy solvent-based high-temperature resistant adhesive system was constructed. By introducing a ferrocene catalyst, the in situ growth of carbon nanotubes (CNTs) at high temperatures was achieved, thereby systematically addressing key issues such as the low room-temperature strength of traditional phosphate-based adhesives, the high-temperature volume shrinkage of resin-based ceramic precursor adhesives, and their mismatched thermal expansion coefficients. This work provided a new technical solution for the reliable high-temperature bonding of C/C composites. The study showed that when the graphene content was 3.2–4% and the number of coating layers was three, the bonding effect was optimal: in the temperature range of 300 °C and below, graphene acted as “bricks” and epoxy resin as “mortar”, achieving high room-temperature strength through π-π conjugation and crosslinking networks, with the bonding strength of both G-4 and G-6 exceeding 10 MPa; when the temperature rose above 700–800 °C, the epoxy resin decomposed gradually, and ferrocene catalyzed the carbon sources in the decomposition products to grow CNTs in situ on the surface of graphene and in the gaps between its layers. These CNTs replaced the resin as a new type of “mortar” and formed a network connection structure with graphene, enabling the G-4 and G-6 adhesives to maintain a bonding strength of more than 3 MPa after treatment at temperatures ranging from 1000 °C to 1300 °C. Additionally, the all-carbon-based structure matched the thermal expansion coefficient of the C/C composite and demonstrated excellent thermal shock resistance. After 15 thermal shocks at 1300 °C, the bonding strength of the G4 and G6 adhesives remained unchanged. The bionic layered reinforcement strategy proposed in this study not only provided a new solution for the high-temperature bonding of non-load-bearing components (such as spacecraft thermal protection tiles and rocket nozzles) of C/C composites but also offered experimental and theoretical support for the application of the “brick-mortar” structure in high-temperature adhesives and the in situ growth technology of CNTs between graphene layers.

## Figures and Tables

**Table 1:** Formulations of different G-X epoxy solvent-borne adhesives.

	Epoxy Solution (g)	Fe(C_5_H_5_)_2_ (g)	Graphene (g)	PEG (g)	SDBS (g)
G-1	25	1	0.6	2	0.025
G-2	25	1	0.4	2	0.025
G-3	25	2	0.6	2	0.025
G-4	25	2	0.8	2	0.025
G-5	25	3	0.8	2	0.025
G-6	25	3	1	2	0.025

## Data Availability

The raw data supporting the conclusions of this article will be made available by the authors on request.
